# Simulation of Gomti River (Lucknow City, India) future water quality under different mitigation strategies

**DOI:** 10.1016/j.heliyon.2018.e01074

**Published:** 2018-12-21

**Authors:** Pankaj Kumar

**Affiliations:** a Natural Resources and Ecosystem Services, Institute for Global Environmental Strategies, Hayama, Kanagawa, Japan; b United Nations University – Institute for the Advanced Study of Sustainability, Tokyo, Japan

**Keywords:** Environmental science, Hydrology

## Abstract

The Gomti River in Lucknow City, India, was an important source of water for the different uses few decades ago. However, because of the rapid global changes, current status of the river is very critical from environmental, aesthetic and commercial usage point of view. Henceforth, this research work focused on assessing the current as well as predicting its future situation using different scenarios while considering key drivers of global changes namely climate change and population growth. Water Evaluation and Planning (WEAP), a numerical simulation tool, was used to model river water quality using two scenarios viz. business as usual (BAU) and scenario with mitigation measures. Water quality simulation was done along 24 km stretch of the Gomti River from downstream of Near Moosa Bird Sanctuary to Near Bharwara. Comparison of simulated water quality parameters for the current and BAU status clearly indicates that the water quality by 2030 will rapidly deteriorate and will be not suitable for many aquatic lives in terms of simulated water quality parameters. Results from scenario with mitigation measures suggest current planned wastewater treatment plants and policies are not sufficient enough to achieve desirable river water quality within class B and hence call for immediate and inclusive action.

## 1 Introduction

Water is the vital natural resource with social and economic value for human beings [1, 2]. At present around the globe, more than 1.1 billion people do not have adequate access to clean drinking water and it is estimated that nearly two-thirds of all nations will experience water stress by the year 2025 [3]. Water security for living needs is threatened not only in terms of water scarcity but also spatio-temporal variation in good quality water, which triggered a change in the focus from water quantity in Millennium Development Goals to water quantity along with quality in Sustainable Development Goals [4]. As Anthropocene is projected to enhance the complexity of water issues around the world, the most prominent factors of global changes are population growth, urbanization and climate change which pose significant challenges to water management and governance [5, 6].

Although water security issues differ across the countries, the most threatening issue is demographic changes along with their life style irrespective of their geographical locations [7]. In addition, for developing countries, weak and non-structured governance simply exacerbate this complex issue of water security rather than the condition of the water resource itself [8]. Recently, different scientific communities are emphasizing on adaptive governance as an important tool for governing social–ecological systems during periods of abrupt global change like Anthropocene. However managing urban water environment is still a challenging task [9, 10, 11]. With swift economic growth and rapid urbanization, it is estimated that about three billion people will live in Asian cities by year 2050 [12] and henceforth, the demands on water, food and other natural resources will be humongous. Renewable water resources in South Asian region have fallen dramatically on a per capita basis since the 1960s and reached the water stressed level for countries like India, Pakistan and Afghanistan by year 2015; whereas approaching rapidly to achieve this water stressed level in near future for many countries like Nepal and Bangladesh [13]. The expected impacts of climate change will further exacerbate the challenges faced by planners and providers of such services. Delivery of sustainable water supply and sanitation services in growing towns and cities remains an issue. Considering the water stress and scarcity, United Nations and its associated members unanimously called for sustainable water resource management to achieve water security through availability of sufficient water with good quality for all as the main agenda of the United Nation Sustainable Development Goals by the year 2030 [14]. So far, authorities for water governance as well as water planning and scientific communities worked in silos especially in developing nations which is a matter of concern. Henceforth, there is an urgent need to integrate both hydrological and socio-economic factors to solve complex issues of water scarcity/security [15]. Better water governance is a must requirement for combating global changes as well as reconciling food security, renewable energy and the provision of multiple ecosystem services [16].

Considering the complex issues of water security, mode of scientific research works gradually evolved from mono-disciplinary research i.e. working in silos for a common goal to transdisciplinary research i.e. working in an integrated way for a common goal. Integrated water resource management (IWRM) modeling tools, as a fine medium for transdisciplinary research targeting at different aspects (socio-economic status, hydro-meteorological factors, agriculture, industries, wastewater etc.) are the need of the hour to come up with scientific evidence based viable water policy decisions [17, 18, 19, 20]. Several IWRM numerical models like RIBASIM (River Basin Simulation Model), MIKE, WEAP (Water Evaluation and Planning), WBalMo (Water Balance Model) have been extensively applied across the globe to solve complex water security issues. Recently, mathematical integrity model was used to analyze the efficiency of localized wastewater treatment plants to handle urban wastewater as well as evaluate the resilience capacity of the water bodies through receiving different residual pollution loads after treatment to help decision makers at city/municipality level both for short and long term strategic mitigation action formulation [21]. Another research work used influence diagram based on DPSIR (drivers-pressures-state-impact-response) framework to evaluate vulnerability of Haihe River basin in China considering climate change, socio-economic exposure and water quality factors and finally suggested potential adaptive measures for the policy makers [22]. Recently the WEAP model has been used widely for water quality modelling and ecosystem preservation because it uses scenario development considering different variables such as industrial activities, climate change population growth, land use/land cover change, status of wastewater treatment plants, sewerage network as well as it is not data intensive and freely available for developing countries [23, 24, 25, 26]. In yet another work, results from IWRM research work was evaluated in terms of the real meaning for the policy oriented choices, institutions and practices, it provides to the policy makers [27]. However, so far most of the IWRM modeling focusses on quantification of water resource quality and quantity using scenario based snapshot of the future world and defining the gaps based on the assumed hypothesis. In doing so, policy based intervention hardly get implemented because of the understanding gap between scientific community and decision makers.

Based on above argument, this study focussed on assessing the current situation and simulated future status of water quality in the Gomti River basin crossing Lucknow city, with ultimate goal to help formulate sustainable water resource management options for the area. Here climate change and population growth were accounted as main driving force behind water quality deterioration and planned wastewater infrastructure from existing master plan as a mitigation measure was considered to predict future water environment. The uniqueness of this research work will be to validate the importance of mitigation measures in the existing master plan for Lucknow city, being developed by the policy/decision makers by considering them in our simulation work for projecting future water environment. In addition, this was a retrofitting process based simulation, where policy makers were consulted while developing the model and after obtaining the primary simulation results for their feedback for the information/data considered for the simulation work. Finally, the obtained results will help them to understand and target the policy gaps necessary to be filled at urgent basis for the sustainable development of water resources. Because this retrofitting process thoroughly involve the decision makers for numerical simulation, they strongly feel ownership of the result and willing to implement the suggested additional measures. The reason to select Lucknow is that; Lucknow is the capital of largest state (Uttar Pradesh) of India, with one of the largest economic centre of the country and witnessing rapid economic/urban growth. Combination of high economic growth and uncoordinated rapid urban expansion results in unhealthy water environment especially around water bodies like the Gomti River basin. Despite its importance, it is very poorly documented for its current status and its management strategies for the near future. Finally, this research work will providing information relevant for the capacity building of the concerned people involved in water planning in right direction.

## 2 Methodology

### 2.1 Study area

**Fig. 1:**
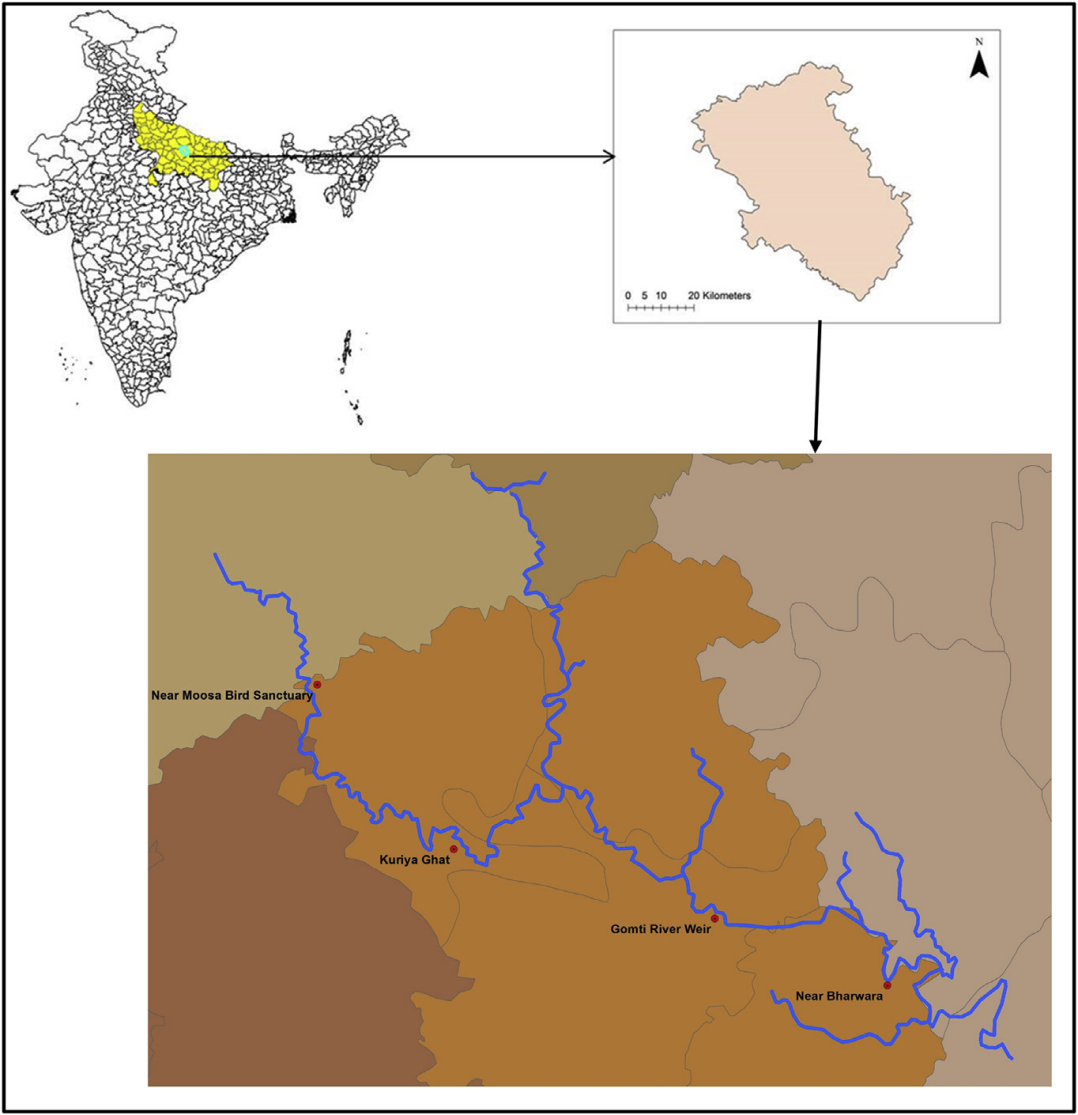
Flow course of the Gomti River passing across the Lucknow City along with few sampling location considered in this study.

Lucknow, the capital of Uttar Pradesh (India), is located in the part of central Gangetic plain between North latitudes 26°30′ and 27°10′ and East longitudes 80°30′and 81°13’ (Fig. 1). The city has a humid subtropical climate with a cool dry winter from December to February and a hot summer from April to June. The temperature extremes varied from 48.9 °C in the summer to 1.67 °C in the winter. The city receives about 900 mm of annual rainfall mostly from the southwest monsoon between July and September. The city's elevation varies from 100 to 130 m above mean sea level and generally slopes to the east. Lucknow is one of the fastest growing city in the country with population projection of 4.7 million in 2031 from 2.8 million in 2011 [28]. Rapid unplanned urbanization has created many problems as it places huge pressure on land, water, housing, transport, health, education etc. This rising population has major impact on natural resources of the area especially on water quality and quantity. Fresh water is the most important natural resources for the life but overexploitation and unjustified use of water has led to deterioration of quality of water.

Lucknow is cut across by a number of streams. Gomti, the major river, flows from North-West to South-East through the center of city. It is one of the major sources of public water supply in the city along with groundwater. Generation of sewage and proper treatment and disposal of this waste is the major problem in the city. Poorly drained sewerage system and lack of treatment capacity of sewage treatment units has resulted in severe degradation the quality of river water.

### 2.2 Basic information regarding about WEAP model

Hydrological simulation with WEAP needs the entire study area to be divided into smaller catchments with consideration of confluence points, physiographic and climatic characteristics. Hydrology module within WEAP can simulate catchment runoff and pollutant transport process within a river/water bodies. To simulate different components of hydrological cycle, WEAP has different catchment methods available namely rainfall runoff (simplified coefficient method), irrigation demands only (simplified coefficient method) and rainfall runoff (soil moisture method). However, for this study, the soil moisture method, the most sensitive method, to estimate the different hydrological components is used. This method can simulate different components of the hydrologic cycle, including evapotranspiration, surface runoff, interflow, base flow, and deep percolation [24]. Here, each catchment is divided into two soil layers: an upper soil layer and a lower soil layer, which represent shallow water and deep water capacities, respectively. The upper soil layer is targeted for spatial variation in different types of land use and soil types, whereas the lower soil layer is considered to represent groundwater recharge and baseflow processes, and its parameters remain the same for the entire catchment. Different hydrological components are estimated, with *z*_1_ and *z*_2_ as the initial relative storage (%) for the upper (root zone) and lower (deep) water capacity, respectively (Eqs. (1), (2), (3), (4) and (5)).

(1) $$ET=Potential\;evapotranspiration*\left(5z_{1}-2z_{2}^{2}\right)/3$$

(2) $$Surface\;runoff=Precipitation\;\left(P\right)*z_{1}^{Runoff\;resistance\;factor}$$

(3) $$Interflow=\left(Root\;zone\;conductivity*preferred\;flow\;direction\right)z_{1}^{2}$$

(4) $$Percolation=Root\;zone\;conductivity*\left(1-preferred\;flow\;direction\right)*z_{1}^{2}$$

(5) $$Baseflow=Deep\;conductivity*z_{2}^{2}$$

z_1_ and z_2_ = upper soil layer and lower soil layer (m), which represent shallow water and deep water capacities, respectively.

The water quality module of the WEAP tool makes it possible to estimate pollution concentrations in water bodies and is based on the Streeter–Phelps model. In this model, two processes govern the simulation of oxygen balance in a river: consumption by decaying organic matter and reaeration induced by an oxygen deficit [24]. The BOD removal from water is a function of water temperature, settling velocity, and water depth (Eqs. (6), (7), (8), and (9)):

(6) $$BOD_{final}=BOD_{\;init}\;\exp^{\frac{-k_{rBOD}L}{U}}$$

(7) $$\text{where}\;k_{rBOD}={k_{d20}}^{1.047\left(t-20\right)}+\frac{\upsilon_{s}}{H}$$

BODinit = BOD concentration at beginning of reach (mg/l), BODfinal = BOD concentration at end of reach (mg/l), t = water temperature (in degrees Celsius), H = water depth (m), L = reach length (m), U = water velocity in the reach, vs = settling velocity (m/s), kr, kd and ka = total removal, decomposition and aeration rate constants (1/time), kd20 = decomposition rate at reference temperature (20° Celsius). Oxygen concentration in the water is a function of water temperature and BOD:

(8) $$Oxygen\;\;saturation\;\;\;or\;\;OS=14.54-\left(0.39t\right)+\left(0.01t^{2}\right)$$

(9) $$O_{final}=OS-\left(\frac{k_{d}}{k_{a}-k_{r}}\right)\left(\exp^{-k_{r}L/U}-\exp^{-k_{a}L/U}\right)BOD_{init}-\left[\left(OS-O_{initial}\right)\exp^{-k_{a}L/U}\right]$$

Ofinal = oxygen concentration at end of reach (mg/l), Oinitial = oxygen concentration at beginning of reach (mg/l).

### 2.3 Data requirement

The WEAP model was used to simulate future water quality variables in the year 2030 to assess alternative management policies in the Gomti River basin. For water quality modeling, a wide range of input data including point and non-point pollution sources, their locations and concentrations, past spatio-temporal water quality, wastewater treatment plants (Central Groundwater Board), population, historical rainfall, evaporation, temperatures (Indian Metrological Department), drainage networks [29], river flow-stage-width relationships, river length, groundwater, surface water inflows, land use/land cover (State water board) and master plan [28] is provided.

**Fig. 2:**
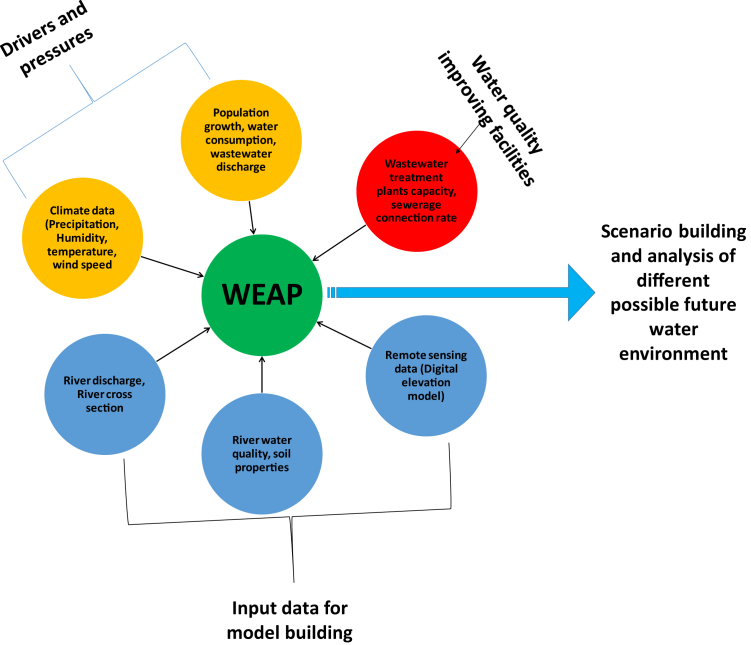
Diagram showing different dataset required to setup the model for this study.

Daily rainfall was collected at IMD Meteorological Station, for the period from 1980 to 2016. Daily average stream flow data in 2011–2016 were measured at five stations, namely Bharwara, River Weir, Pipraghat, Kuriyaghat, Manjhighat and Near Moosa Bird Sanctuary collected from Indian Meteorological Department and were utilized to calibrate and validate the WEAP hydrology module simulation. Data for the water quality indicators biochemical oxygen demand (BOD) and total coliforms (which was later assumed as equivalent to *Escherichia coli* counts) were also collected at above all five stations, which were used for water quality modeling. All dataset required to build the model and scenario analysis for this study is shown in Fig. 2.

**Fig. 3:**
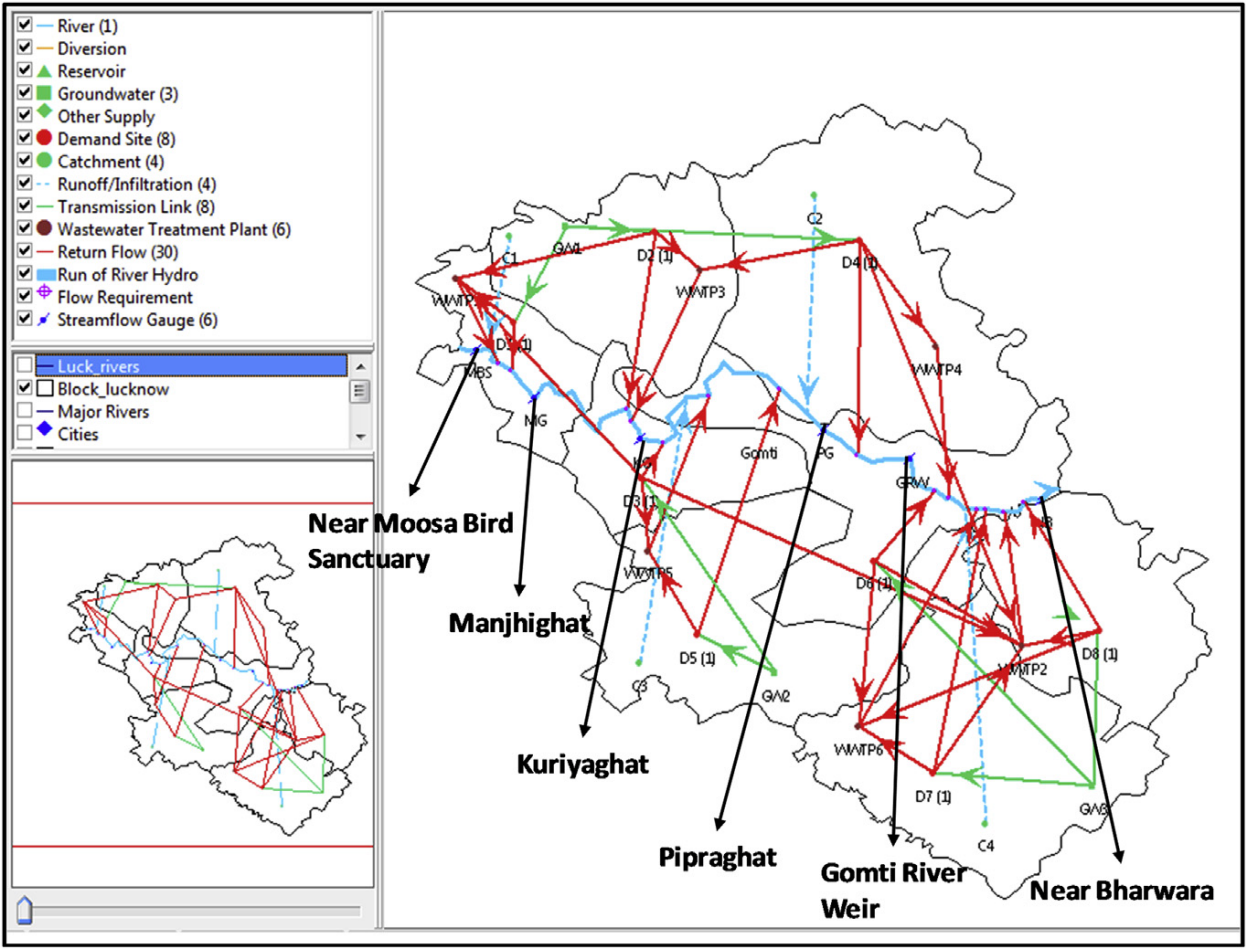
Schematic diagram showing the problem domain for water quality modeling in Gomti River using WEAP interface.

The WEAP model was developed for the Gomti River basin for four catchment areas with inter-basin transfers with consideration of the confluence points and physiographic and climatic characteristics (Fig. 3). Pollutant transport from a catchment accompanied by rainfall-runoff is enabled by ticking the water quality modeling option. Pollutants that accumulate on catchment surfaces during non-rainy days reach water bodies through surface runoff.

Regarding future precipitation data, different Global Climate Models (GCMs) and Representative Concentration Pathway (RCP) output were used after downscaling and bias correction. In order to evaluate the climate change on water quality, we have evaluated the change in monthly average precipitation. Statistical downscaling followed by trend analysis, a less demanding computation technique which enables reduction of biases in the precipitation frequency and intensity [30] is used here to get climate variables at monthly scale. Historical rainfall analysis using the monthly precipitation data of 25 years (1980–2004) was done. Understanding the potential impacts of climate change is essential for informing both adaptation strategies and actions to avoid dangerous levels of climate change. This study carried out a comprehensive assessment of the possible climate change over study area by using MRI-CGCM3 and MIROC5 as GCMs with RCP4.5 and RCP8.5 emission scenarios. Future climate corresponds to the period of 2020–2044.

In order to estimate the effect of population growth on water quality status, the whole study area is being divided in to eight demand sites. These demand sites mainly represent the population of different cities lying on either side of the Gomti River within our study. Location of demand sites were chosen in such a manner that it will a give a symmetric representation of pollution load (in terms of domestic discharge) from the inhabitants living at both sides of the river banks. Result for the population distribution and its future trend at these demand sites were calculated by ratio method using UNDESA projected growth rate [12].

Apart of above mentioned components for model setup, other major considerations are the eight demand sites and two wastewater treatment plants (WWTPs) to represent the problem domain. Here, demand sites are meant to identify domestic (population) centers defined with their attributes explaining water consumption and wastewater pollution loads per capita, water supply source and wastewater return flow. Dynamic attributes are described as functions of time and include population. Wastewater treatment plants are pollution handling facilities with design specifications that include total capacity and removal rates of pollutants. Also, sewerage connection rate for households in the study area was assumed as equals to percentage of total domestic waste water being transported to the WWTPs. In other words, clogging and leakage of sewerage pipeline was neglected. In this study, domestic sewerage is only source considered for incoming wastewater into the Gomti River and its tributaries.

Here, upflow anaerobic sludge blanket reactor coupled with sequencing batch reactor (UASB-SBR) type of wastewater treatment plant is considered in the modeling and its treatment efficiency is assumed as 97% for BOD and 99.69% for fecal coliform [31]. Type and specification of the currently existing WWTPs was not available in any report. However, for the WWTPs to be built in future, type and specification will be of UASB-SBR technology as mentioned in the existing city master plan for year 2030 [28]. Henceforth, for the simplicity, UASB-SBR technology was adopted both for current and future WWTPs during the simulation. No precise data are available regarding the total volume of wastewater production from domestic sources. In the absence of detailed information, the daily volume of per capita domestic wastewater generation considered for this study was 130 liters [32]. Once model set up is done, first calibration then followed by validation is done using simulated result of water quality for current situation i.e. 2015 and average river discharge for three months for years 2013–2015. Thereafter, numerical simulation is conducted using different scenarios called business as usual (BAU) scenario and scenario with mitigation measures. For current and BAU scenario, WWTP capacity was 145 MLD (total number = 2), whereas for scenario with mitigation measures this capacity was 1119 MLD and total number of WWTPs was 9 [28]. There can be several possible mitigation measures for improving water environment like nature based solution, people awareness, decentralized wastewater treatment plants etc. But for this study, city master plan was only considered where structural measures with increasing capacity of WWTPs and sewerage connection rate as the only foreseeable mitigation measures is mentioned. In addition, government is putting lot of efforts on priority basis to accomplish this target within stipulated time i.e. 2030. Different projects in this regard are on progress or for some projects financial grants are already approved. Therefore, this study wanted to show the effect of increasing WWTPs capacity and sewerage network improvement as a structural measures to improve water environment and suggest the decision makers with different gaps if any, so that they can take timely action.

## 3 Results and discussion

### 3.1 Precipitation change

**Fig. 4:**
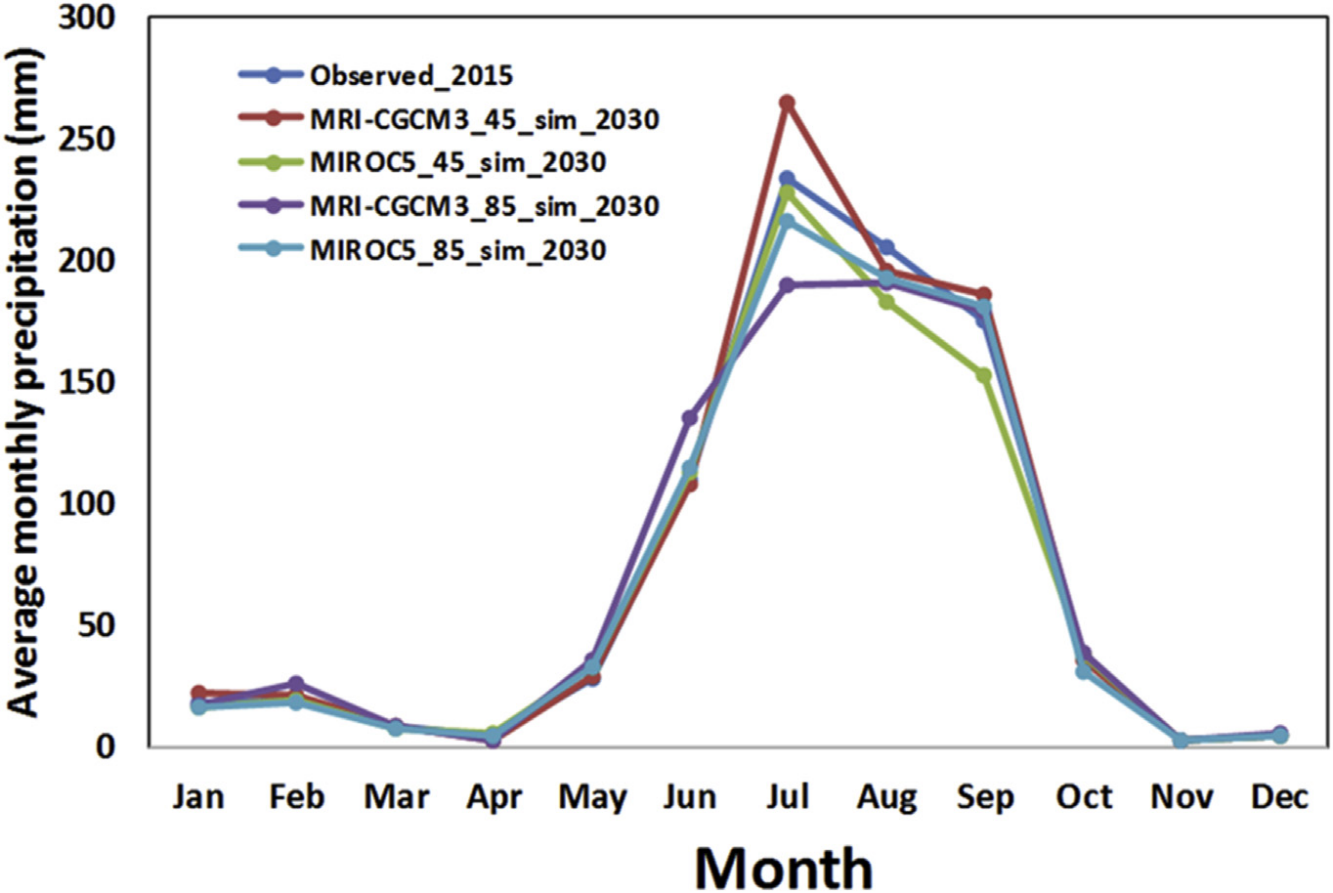
Graph showing a comparative study for current and future monthly rainfall at IMD station.

The comparative results of the monthly precipitation pattern are shown in Fig. 4. The observed annual precipitation for the years 2015 was 844.8 mm. On the other hand, the projected annual precipitation for the year 2030 using MRICGCM3 GCM under RCP 4.5 and RCP 8.5 was 883.1 mm and 831.8 mm respectively. On the other hand, the same projected precipitation value using MIROC5 GCM with RCP 4.5 and RCP 8.5 was 802.0 mm and 822.1 mm respectively. These values clearly indicates that annual precipitation in the simulation from GCM output is not much different than that of current observed one.

### 3.2 Population growth

The total population of 4589838 was considered at base year i.e. 2011 in our study area [28]. For the future population projection, the percentage annual growth rate was considered 2.42, 2.24, 2.26 and 2.16 during the period of 2011–2015, 2016–2020, 2021–2025, and 2025 to 2030 respectively [12]. Henceforth, the population considered for current year (2015) and target year (2030) was 5050525 and 7020597 respectively.

### 3.3 Water quality

#### 3.3.1 Model performance evaluation

**Fig. 5:**
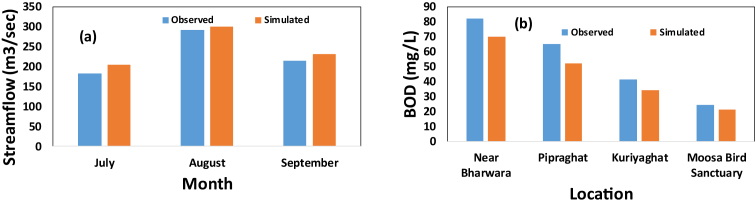
Validation of the model output by comparing simulated and observed (a) average monthly river discharge for year 2012–2014 at Pipraghat; (b) Average BOD values for different locations for the year 2014.

Before doing future scenario analysis, performance of the WEAP simulation is calibrated and validated with observed and simulated values of hydrological and water quality parameters. Hydrology module parameters (effective precipitation and runoff/infiltration) were adjusted using trial and error method during simulation in order to reproduce the observed monthly stream flows for the period of year of 2013–2015 in case of hydrology module validation (Table 1). The final best fit parameter for both entities was 93% and 60/40 respectively. Fig. 5(a), compares monthly simulated and observed stream flows at Pipraghat (average value for year 2012–2014), showing that they largely match for most months with correlation coefficient (R2) ≅ 0.80, root-mean-square error (RSME) ≅ 0.25, and an average error of 10%. The reason to select the three months for validation is because there is no water available in the river especially during dry period. Whereas, water quality simulation part is validated by comparing yearly average simulated and observed BOD concentration for the year 2014 at different locations stretching from upstream to downstream. Selection of this location and time i.e. year 2014 was made on the basis of consistent availability of observed water quality data. Results show a strong relation between these two (Fig. 5(b)) (with error of 12%) confirming suitability of the model performance in this problem domain.

**Table 1:** Summary of parameters and steps used for calibration.

Parameter	Initial Value	Step
Effective precipitation	100%	±0.5%
Runoff/infiltration ratio	50/50	±5/5

#### 3.3.2 Scenario analyses

For water quality, simulation is done using two possible scenarios as shown in Table 2. Simulation was done for the years 2015 and 2030 using 2011 as reference year with consideration for population increase, wastewater generation and its treatment at waste water treatment facilities. Once model was validated, water quality parameters were simulated at monthly scale for the year 2015 i.e. current situation. Thereafter, for business as usual scenario, effect of climate change and population growth was observed for year 2030, while keeping wastewater infrastructure constant similar as to that of the current situation i.e. WWTPs capacity of 145 MLD. Here to evaluate effect of climate change, average values of rainfall and temperature from two GCMs with two RCPs was considered. For the scenario with mitigation measures, all conditions were kept the same except increased wastewater plant capacity and collection rate as mentioned in Table 2.

**Table 2:** Summary of all the criteria considered for different scenarios in future water quality simulation.

Scenario	Components
Business as usual	Climate change + population growth + WWTP of 145 MLD (19% collection rate)
With mitigation measures	Climate change + population growth + WWTP of 1119 MLD (100% collection rate)

**Fig. 6:**
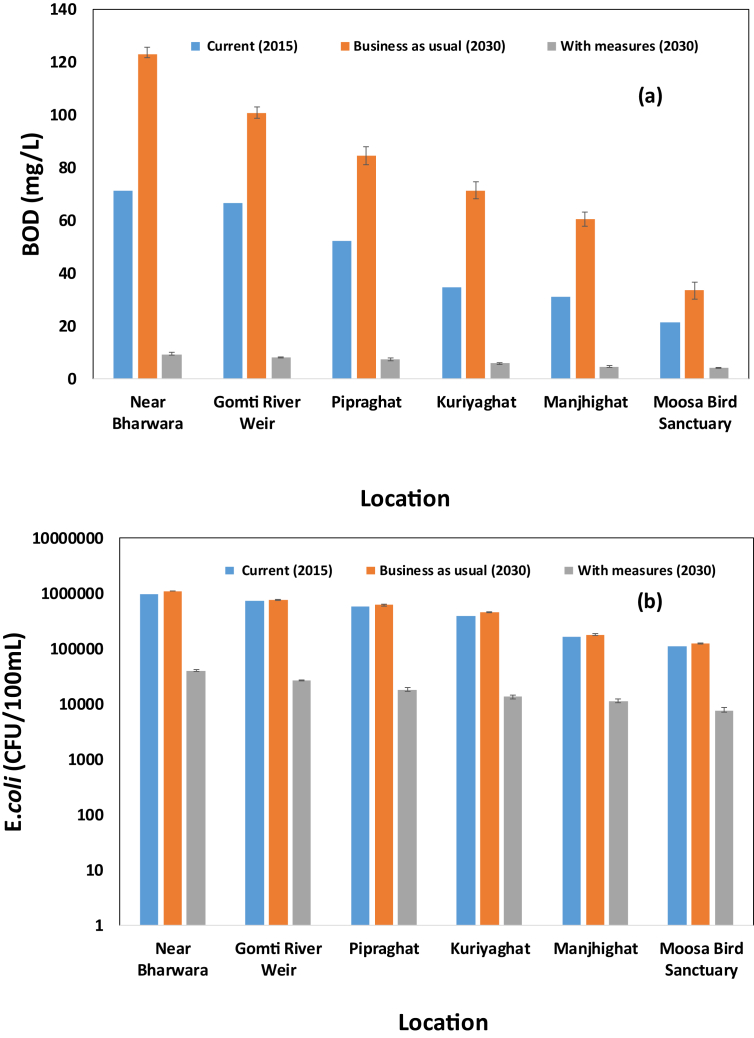
Result shows simulated water quality parameters (a) BOD (b) *E. coli* for different scenarios (Here Business as usual scenario considering population growth and average climate change and WWTP of 145 MLD capacity where as scenario with measures considering population growth and average climate change and WWTP of 1119 MLD capacity).

The simulation results for the water quality parameters (BOD and *E. coli*) using these two scenarios is shown in Fig. 6. Here, small bars on the simulated water quality show the range due to change in GCM and RCP. With the currently existing wastewater treatment plant (Capacity of 145 MLD and mere coverage of 19% of total population in the study area), the current status of water quality throughout the river is very poor if compared with local guideline for class B i.e. swimmable category (BOD<3 mg/L and *E. coli*<1000CFU/100mL) [33]. For the year 2015 (current stage), the simulated value of BOD varies from 21.5 to 71.4 mg/L, which clearly indicates that all the water samples are moderately to extremely polluted compared with class B. Looking into the result from scenario 1 i.e. business as usual, the effect of both climate and population changes are prominent in water quality status. It is deteriorating further in 2030 when compared to the current situation. Due to both climate and population changes, the water quality expressed as BOD and *E. coli* will be deteriorating by a further 70.8% and 10.6 % respectively on an average in 2030 when compared to the current situation. Further, the effect of each individual parameter i.e. population growth and climate change is analyzed by keeping other parameters constant. For example, when calculating the individual effect of population growth, the value of rainfall as a representative of climate change in this case by year 2030 kept constant and vice-versa. The obtained result is shown in Table 3, where it is very clear that population growth has bigger contribution in water quality deterioration to climate change.

**Table 3:** Summary of the effect of individual parameters on simulated water quality.

Parameters	Average % increase with business as usual scenario (2015–2030)	% Contribution from population growth	% Contribution from climate change
BOD	70.8	88	12
*E. coli*	10.6	90	10

However, based on scenario with measures, where whole domestic wastewater generated within the study area will be collected by 100 % sewerage collection rate and treated from increased WWTPs capacity of 1119 MLD, result for simulated water quality will be much better. The concentration of BOD and *E. coli* during scenario with measures will be reduced by 91.7% and 96.4 % respectively compared to the business-as-usual scenario throughout the stream, which is an encouraging sign. However, quality is still a matter of concern especially in the downstream area when compared with class B. The main reason behind inability of this mitigation measure to improve water environment within the desirable limit, was non-consideration of the effect of climate change, one of the potential driver for future water resources deterioration, in the current master plan. Although effect of climate change is numerically small as evident from this study, it is very important to consider these factors for making existing master plan more robust at long time scale. The above result suggests that current management policies and near future water resources management plan are not enough to check the pollution level within the desirable limit and calls for transdisciplinary research in more holistic way for doing it sustainably. Also, from the health risk point of view, this simulated water quality result shows potential risks like gastroenteritis once consumed accidently (microbial contamination), algal bloom and death of aquatic organisms like fishes (because of high BOD).

**Fig. 7:**
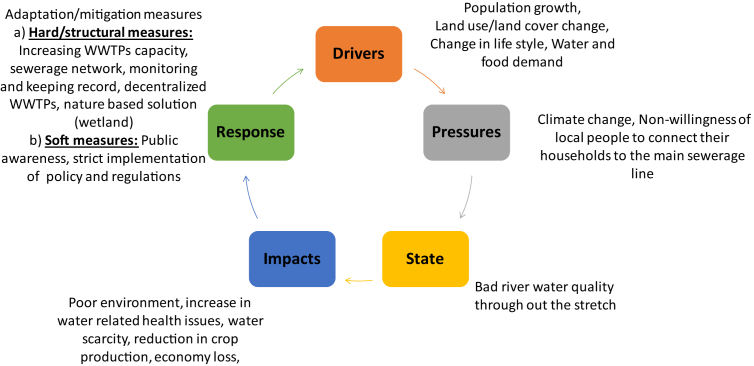
DPSIR framework for investigating impacts of poor wastewater management on future water environment and their possible countermeasures.

Finally, the logic of Drivers-Pressures-State change-Impact-Response (DPSIR) framework (Fig. 7) is used to present summary of key finding from this research work. Because of different key drivers (population growth, land use/land cover change, life style change) and pressures (climate change, non-willingness to connect to main sewerage lines), water resources (quality as well as quantity) is in highly vulnerable condition. This led to sharp depletion of fresh water for both quantity as well as quality. Although this is a common global trend, however the worst affected regions are developing or undeveloped countries with limited resources/infrastructure and weak governance. This poorly managed water resources results in poor aesthetic/environmental condition, loss of economy and increase in water related health issues. For such complex issues of water security, there is a need of integrated top-down as well as bottom-up approach. Which means, not only a better efforts from policy makers at national/regional level but also participatory approach from the local people is very much needed. We first need to target the source of pollution and minimize it, for which both hard/structural measures (increasing WWTPs capacity, sewerage network, artificial wetlands) and soft measures (people awareness about benefits of good water environment, strict implementation of policy and regulation regarding managing water resources at watershed/basin level) are required. Since financial resources is one of the most important limiting factor for such mitigation/adaptive measures, it is very important to prioritize different actions and look for different potential donors like Asian Development Bank, World Bank etc. and mechanisms like CDM (Clean Development Mechanism).

## 4 Conclusion

This work gave a detailed picture of water quality of Gomti River in Lucknow City, India for both current (2015) and future (2030) time using different scenario analyses. Simulated result clearly indicated that Gomti River is moderately to severely polluted throughout the stretch when compared with class B given by Uttar Pradesh Pollution Control Board (UPPCB) for year 2015. In addition, for the business as usual scenario, the quality status will become worse by year 2030. However, considering the scenario with mitigation measures as mentioned in local master plan for water resources management, the quality of water will improve significantly. However, water quality at downstream areas like Pipraghat, Gomti River Weir and Near Bharwara does not comply with desirable water quality of class B, and needs further attention. Some of the other potential reasons behind the poor status of water quality are: a) at current stage, despite the considerable capacity of existing WWTPs, the wastewater coming to these plants are not sufficient because of poor sewerage collection rate or poor connection between each household and main sewerage line. The reason behind this is the non-willingness to pay the expensive connection fee by the local residents; and even once, connected they have to pay more money in terms of water or sewerage treatment bills; b) lack of proper coordination between different actors/stakeholders involved in water management to implement the master plan (water infrastructure) in a timely manner.

Possible solutions to overcome these barriers can be: a) to create a political space where different stakeholders other than government agencies also have direct involvement to influence the governing processes and government decisions; b) provision of some monetary help like financial incentives in terms of tax exemption to encourage local people to connect there households to the main sewerage line; c) raising people awareness about the benefits of better water environment in terms of good health, good business opportunities by tourism; d) as creating different hard measure (wastewater treatment) is financial burden for many developing countries and that's why many projecting are running behind their scheduled progress rate. Henceforth, there should be more push to the local government to implement de-centralized WWTPs along with centralized WWTPs as other possible mitigation measure which is subject of future research; e) in order to complete the exiting master plan by the given timeline, regular monitoring for progress of implementation of master plan is highly recommended; f) consider more precise information for various pressures and drivers for global changes namely population density, population growth, climate change, land use/land cover change in the revised master plan for achieving desired future water environment both at short and long time scale.

## Declarations

### Author contribution statement

Pankaj Kumar: Conceived and designed the experiments; Performed the experiments; Analyzed and interpreted the data; Wrote the paper.

### Funding statement

This research did not receive any specific grant from funding agencies in the public, commercial, or not-for-profit sectors.

### Competing interest statement

The authors declare no conflict of interest.

### Additional information

No additional information is available for this paper.
